# Double-Network Hydrogel with Tunable Mechanical Performance and Biocompatibility for the Fabrication of Stem Cells-Encapsulated Fibers and 3D Assemble

**DOI:** 10.1038/srep33462

**Published:** 2016-09-15

**Authors:** Zhe Liang, Chenguang Liu, Lili Li, Peidi Xu, Guoan Luo, Mingyu Ding, Qionglin Liang

**Affiliations:** 1 Department of Chemistry, Tsinghua University, Beijing, 100084, China

## Abstract

Fabrication of cell-encapsulated fibers could greatly contribute to tissue engineering and regenerative medicine. However, existing methods suffered from not only unavoidability of cell damaging conditions and/or sophisticated equipment, but also unavailability of proper materials to satisfy both mechanical and biological expectations. In this work, a simple method is proposed to prepare cell-encapsulated fibers with tunable mechanical strength and stretching behavior as well as diameter and microstructure. The hydrogel fibers are made from optimal combination of alginate and poly(N-iso-propylacrylamide)-poly(ethylene glycol), characteristics of double-network hydrogel, with enough stiffness and flexibility to create a variety of three dimensional structures like parallel helical and different knots without crack. Furthermore, such hydrogel fibers exhibit better compatibility as indicated by the viability, proliferation and expression of pluripotency markers of embryonic stem cells encapsulated after 4-day culture. The double-network hydrogel possesses specific quick responses to either of alginate lyase, EDTA or lower environmental temperature which facilitate the optional degradation of fibers or fibrous assemblies to release the cells encapsulated for subsequent assay or treatment.

Fabrication of cell-encapsulated fibers is one of the hottest emerging topics on tissue engineering and regenerative medicine as the potential to be used as fundamental components^1^,^2^,^3^. Traditional fabricating methods of fiber-shaped constructs include electrospinning^4^,^5^, wetspinning^6^,^7^ and microfluidic spinning^8^,^9^. Nanoscale fiber-based material with divergent shapes and sizes made by electrospinning have the possibility to be widely used in manufacture biomimetic scaffolds as it provides microstructure that similar to native constructs^10^. Unfortunately, cells are usually seeded onto the surface of electrospinning matrix, otherwise serious damages are inevitable by the high voltages applied during the process. Wetspinning and microfluidic spinning could offer much milder conditions and more opportunities in construction design. Since its proposal, microfluidic technology has become spotlight in many fields because of the capacity of precisely control over fluidic processes^11^. Complex-shaped fibers were fabricated using template-aided multiphase flow based on polymeric jet streams and *in situ* photopolymerization^12^. Microfluidic chips with hierarchical, multilayer and channel structures were manufactured in order to form hydrogel fibers with different structures^13^,^14^. Nonetheless, residues derived from the immiscible solvent as well as the lithography process may cause cytotoxicity and well-trained specialists are needed to operate the sophisticated equipment. Therefore, the requirement for a simple, versatile, and low-cost system for the fabrication of cell-laden fibers is urgent.

Another challenge which limited the final application of cell fibers is the matrix. The vital role of scaffolds in tissue engineering is providing native-mimicking environment for cells proliferation, differentiation and regeneration^15^. Although native-derived hydrogel such as collagen, matrigel and fibrin have good biocompatibility and biodegradability^16^,^17^, they are not suitable for tissue engineering due to their limited mechanical strength. Alginate is one of the most widely used Ca^2+^-triggered natural derived hydrogel which can provide satisfying mechanical strength^18^,^19^ while lack of moieties for ligand binding. On the other hand, synthetic hydrogels usually hold the merits of great mechanical performance, designable molecular structure, and responsiveness to external stimulus. Stimuli-responsive polymers, such as GelMA^20^, PHEMA^21^, PNIPAM^22^,^23^, and DNA hydrogel^24^ are considered promising biomaterials in microfabricating as they possess responsiveness to external environmental perturbations. The biocompatibility of most of synthetic materials is unsatisfactory^25^ Besides, cell damaging often occurred during the cross-linking procedures like irradiation under UV light^26^. Among massive thermo-responsive polymers, copolymer of poly(N-iso-propylacrylamide) and poly(ethylene glycol) (PNIPAAm-PEG) is well-suited for cell culture for the following reasons. (1) PNIPAAm-PEG is a thermo-reversible polymer that shows liquid state at low temperature and solidifies into elastomeric hydrogel when warmed up. Cells can be encapsulated into hydrogel at 4 °C on ice, cultured in incubator at 37 °C, while released back on ice or in refrigerator if needed. Transition temperature is moderate to cells and is easy to manipulate. High temperature explosion can be avoided. (2) The highly lipophilic environment recapitulate features of the natural extracellular matrix which could accelerate cell proliferation and communication, as well as protect cells from shear stress. It has been proved that PNIPAAm-PEG holds much better cell compatibility comparing to other synthetic materials, even some native derived ones^27^. However, the poor mechanical performance limits its application in biofabrication. To summarize, no one single polymer meets all the requirements that are essential in tissue engineering. Thus, creating a reinforced double-network hydrogel (DNH) combining the advantages of both natural-derived and synthetic hydrogels may be a possible strategy to solve the problem.

To address these issues, in this work we proposed a simple method to prepare cell-laden DNH fibers made of PNIPAAm-PEG and alginate with tunable stiffness and flexibility. Alginate here serves as a bracket providing mechanical strength for handling, while PNIPAAm-PEG accelerates cell adherence and proliferation. The resulting system has the ability to form mechanically stable, porous, hydrated three dimensional network. These DNH fibers can be assembled into a variety of three dimensional constructs and cells encapsulated can be released through various pathways, among which a non-chemical adding method is highlighted. The availability, biocompatibility and degradability make it attractive for numerous biomedical applications, particularly in fields of tissue engineering and regenerative medicine.

## Results

### Fabrication and Characterization of DNH Fibers

**Figure 1:**
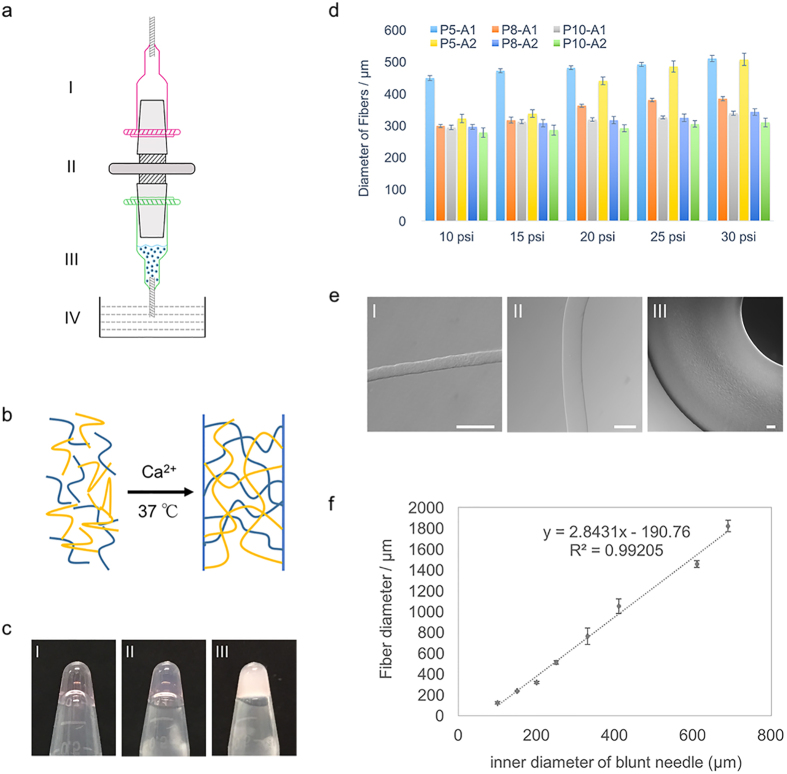
Formation and characterization of DNH fibers. (**a**) Schematic of the critical part in microfluidic pneumatic dispensing system. (I) blunt needle, (II) luer connector, (III) cells encapsulated in hydrogel, (IV) CaCl_2_ and sucrose solution. (**b**) Schematic of the crosslinking method. (**c**) Images of (I) 1% alginate (II)10% PNIPAAm-PEG (III) corresponding DNH in E8 medium. Freshly prepared DNH solution was turbid whereas the pure alginate and PNIPAAm-PEG were optically transparent. (**d**) Diameter of DNH fibers prepared using 27G needles under different pressures. The diameter showed a positive correlation with pressure while a negative correlation with the concentration of both components. (**e**) Fibers fabricated using the microfluidic pneumatic dispensing system with diameters ranging from 40–2000 μm. Scale bar 200 μm. (**f**) The linear correlation between the practical diameters of the DNH fibers and the inner diameters of corresponding blunt needles. The results are illustrated as the mean ± s.d. of ten independent fibers.

Fibers were prepared using microfluidic pneumatic dispensing system. The schematic diagram is shown in Fig. 1a. PNIPAAm-PEG and alginate were mixed thoroughly on ice before loading into the stub. Fourier transform infrared (FTIR) spectroscopy images (Supplementary Fig. S1) showed no covalent bonding between the two components. After resetting the device, air pump was turned on to extrude fibers into warm CaCl_2_ solution. Once extruded into warm CaCl_2_, the pre-gel hydrogel went through sol-gel transition immediately, triggered by Ca^2+^ and temperature at the same time. Unlike methods which need several steps to crosslink, this process can be finished in one-step. Figure 1b illustrates the fast gelation procedure. As shown in Fig. 1c, the freshly prepared DNH solution is turbid whereas pure alginate and PNIPAAm-PEG are optically transparent. Cells were fixed into hydrogel fibers during the procedure if they were mixed into pre-gel solution at the beginning.

Diameter of fibers was influenced by different air pressure and formulation. Briefly, DNH with different mixing ratio of PNIPAAm-PEG and alginate were labelled as (P-A), where P and A indicate the concentrations of PNIPAAm-PEG and alginate in wt/vol % respectively. Six formulations (P5-A1) (P8-A1) (P10-A1) (P5-A2) (P8-A2) (P10-A2) were prepared, as well as the pure two components A1, A2, P5, P8 and P10. As demonstrated in Fig. 1d, diameter has a positive correlation with pressure ranging from 10–30 psi while a negative one with the concentration of both components. 27G needles (inner diameter 200 μm) were used. This phenomenon can be explained by the relationship between volumetric flow rate (*Q*) and crosslinking rate. *Q* is determined by the Poiseuille law as equation (1)


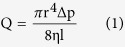


where *r* is the radius and *l* is the length of pipe, *η* is the viscosity of solution and *∆p* is the pressure loss between the two ends. Crosslinking rate and *η* are constant when the formulation is fixed. *Q* has positive correlation with *∆p*. When the crosslinking rate is much lower than that of extrusion (determined by *Q*), the pre-gel solution tended to diffuse in CaCl_2_ solution, which results in a larger diameter. In another case, when *∆p* is fixed, *Q* has a negative correlation with *η*. Thicker fibers are available by decreasing the proportion of PNIPAAm-PEG and/or alginate. Thus, fibers with stable and tunable sizes can be obtained by varying pressure, formulation and specification of needles. Theoretically, the length of the fiber is infinite.

By altering the specification of blunt needles, fibers with diameters ranging from 40–2000 μm have been made (Fig. 1e), which almost cover the scale of fiber-shaped functional tissues *in vivo*^1^. Figure 1f shows the linear relationship between the practical diameter of DNH fibers and the inner diameter of different blunt needles. It was found that because of extrusion-crosslink solidification, the practical diameters of the DNH fibers were larger than the inner diameters of corresponding blunt needles. As shown in Fig. 1f, the linear equation was y = 2.8431x–190.76, R^2^ = 0.99205. As the practical diameter can be calculated by the equation, DNH fibers with various target sizes can be obtained by simply changing specification of blunt needles. Connectors used here are all standard elements. Capillaries or three-dimensional printing components are not necessary comparing to other complicated methods.

**Figure 2:**
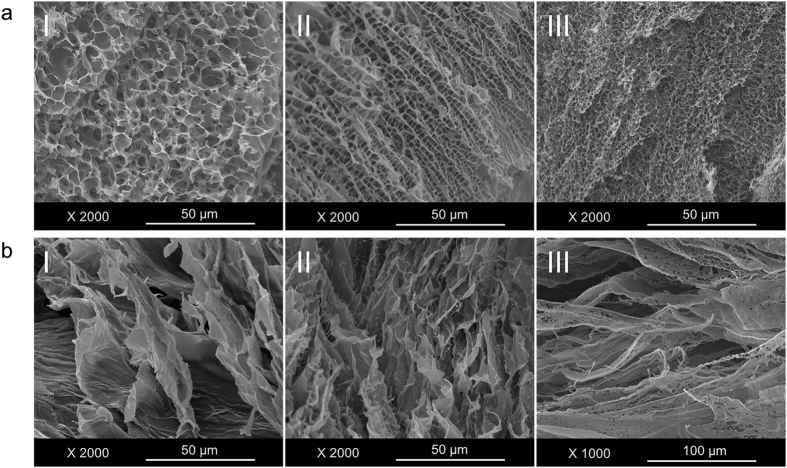
Scanning electron microscopy images of different hydrogels. (**a**) SEM images of DNH at different ratio. (I) P5-A1, (II) P8-A1 and (III) P10-A1. Compared with pure components, the reinforced double network material was highly porous. By altering the mixing ratio, hydrogel with different pore sizes in a certain range like several microns to twenty microns can be obtained. (**b**) SEM images of (I) pure alginate, (II) pure PNIPAAm-PEG and (III) gel matrix after selective sacrificing the PNIPAAm-PEG component in the DNH. Alginate showed lamellar structure while PNIPAAm-PEG was more wrinkled. Massive pores showed up on the lamellar structure owing to the sacrifice of PNIPAAm-PEG.

To investigate the microstructure of DNH and the two components, pure PNIPAAm-PEG, pure alginate and DNH with different proportion were observed under scanning electron microscope (SEM). As shown in Fig. 2a, the reinforced DNH is highly porous. Pore sizes decreased with increasing PNIPAAm-PEG at fixed proportion of alginate. By altering the mixing ratio, hydrogel with different pore sizes in a certain range like several microns to twenty microns can be obtained. Morphology of PNIPAAm-PEG and alginate were totally different (Fig. 2b). Alginate showed lamellar structure while PNIPAAm-PEG was more wrinkled. Comparing with the two components, DNH are more porous. The arranged pores could guarantee enough space for cell proliferation, exchange of nutrients and metabolic wastes. Thus DNH is more suited for cell culture (will be demonstrated in part 2.4.) To further clarify the combining behavior of the DNH, SEM was used to investigate the morphologies of loose gel matrix made of only alginate after selective sacrificing PNIPAAm-PEG. As illustrated in Fig. 2b, the morphology of the gel matrix after selective sacrificing the PNIPAAm-PEG component is very different from pure alginate. Massive pores showed up on the lamellar structure owing to the sacrifice of PNIPAAm-PEG. This phenomenon proved that PNIPAAm-PEG and alginate were not intermolecular-dependent in the double-network system.

### Mechanical Properties of Hydrogel Fibers

**Figure 3:**
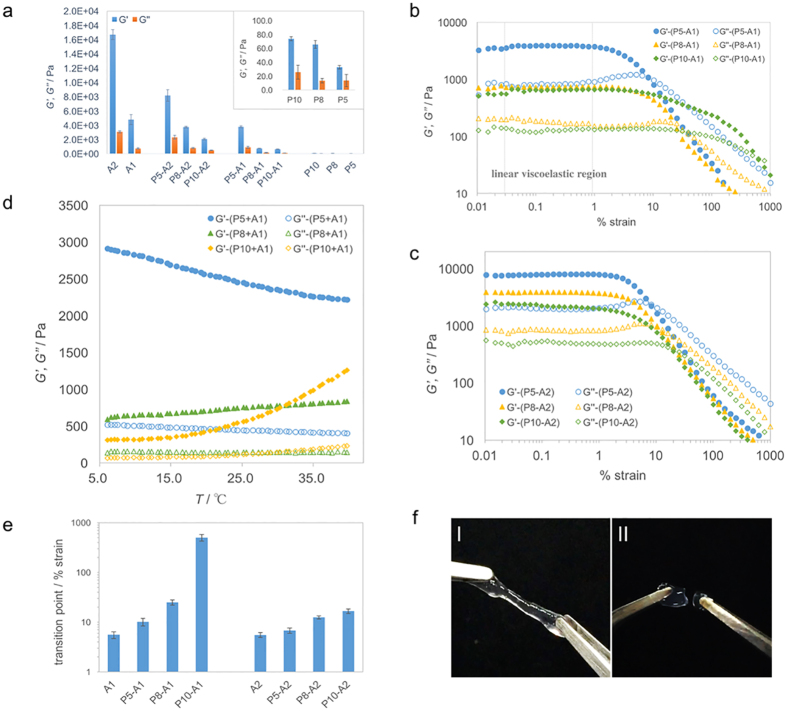
Mechanical properties of hydrogel fibers. (**a**) Rheological characterization of DNH were tested. Average G’ and G” values were recorded at a fixed frequency (1 Hz) and strain (0.5%) at 25 °C. Mechanical strength of DNH with different proportion of PNIPAAm-PEG with a fixed concentration of alginate at (**b**) 1% or (**c**) 2%. Frequency and temperature were fixed at 1 Hz and 25 °C respectively. (**d**) Temperature-ramp rheological analysis was tested from 4 to 40 °C, temperature ramp rate of 4 °C min^**−1**^, fixed frequency (1 Hz) and strain (0.5%). (**e**) Sol-gel transition point of different hydrogel. Sol-gel point increased along with the addition of PNIPAAm-PEG, which means the stretching behavior of alginate had been improved tremendously. (**f**) Stretching process of 100 μL (I) P10-A1 and (II) A1. The results are illustrated as the mean ± s.d. of three independent samples.

In order to evaluate the mechanical property of DNH with different formulations, rheological experiments were carried out on an AR-G2 rheometer equipped with temperature controller. Mechanical strength of (P5-A1) (P8-A1) (P10-A1) (P5-A2) (P8-A2) (P10-A2), pure alginate and pure PNIPAAm-PEG were examined at room temperature. Results are listed in Fig. 3. In all samples, G’ values were found to be significantly higher than G” over a broad range of frequency, indicating that they indeed formed gel states as desired. As demonstrated in Fig. 3a, higher proportion of PNIPAAm-PEG induced lower G’ value when the concentration of alginate is fix. Alginate had opposite effect on G’ value on the other hand. G’ increased from 654.8 Pa of (P10-A1) to 7662.6 Pa of (P5-A2). Linear viscoelastic regions are illustrated in Fig. 3b,c. By altering the formulation, stiffness of the DNH can be tuned. While traditionally employs various growth factors, stem cell differentiation has become increasingly linked to the mechanical property of matrix^28^. As the mechanical property of cell niches varies from 0.1 kPa of soft brain tissue to more than 30 kPa of the rigid calcifying bone^29^, adjustability of the matrix stiffness may help guide cells during differentiation processes. Different from most double-network systems, the resulting matrix didn’t exhibit higher mechanical properties than alginate alone. We deduced that the incorporating PNIPAAm-PEG interfered the crosslinking process of alginate, reducing the actual amount the DNH. In order to verify this speculation, relative amount of Ca in A1 and P10-A1 were measured using Inductively Coupled Plasma Optical Emission Spectrometer (ICP-OES). Degree of crosslinking is converted and calculated as mentioned in Supplementary Method 1. Results revealed that the crosslinking degree of alginate in P10-A1 was only 49.9% while 78.9% in A1, confirming the speculation mentioned above. With larger amount of PNIPAAm-PEG, viscosity increased and pore size decreased (Fig. 2). We hypothesize that these factors simultaneously hindered the diffusion efficiency of Ca^2+^, reducing the actual amount of cross-linked alginate in DNH, which ultimately led to the decrease of mechanical strength.

Further study of the thermal behavior was performed among hybrid gels. Data are shown in Fig. 3d. In the case of P10-A1, G’ increased significantly with temperature from 4 °C to 40 °C. P8-A1 had similar trend with P10-A1 albeit more slowly. As to P5-A1, the effect of PNIPAAm-PEG cannot compensate that of alginate, thus it showed a downward trend similar with that of alginate (Supplementary Fig. S2). Stretching behavior were also illustrated in rheological data. Sol-gel transition point (the intersections of G’ and G” curve) was confirmed to be in positive correlation with concentration of PNIPAAm-PEG and negative with that of alginate (Fig. 3e). The sol-gel transition points reached 505.69% of P10-A1 while merely 5.59% of A1. The flexibility of alginate had been improved tremendously because of the addition of PNIPAAm-PEG. As shown in Fig. 3f, hydrogel P10-A1 can be uniformly stretched up without fracturing at room temperature. On the contrary, pure alginate is too brittle to be stretched and pure PNIPAAm-PEG cannot be handled. These phenomena also indicated that DNH overcome both the brittleness of alginate and low mechanical strength of PNIPAAm-PEG.

### Three Dimensional Assemble by Hydrogel Fibers

**Figure 4:**
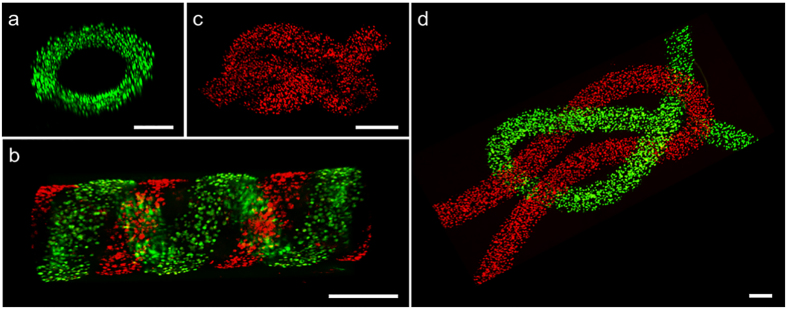
Fiber-based assemble of three dimensional structures. Confocal images of (**a**) helical tube, (**b**) parallel helical tube, (**c,d**) different knots. Cells were labeled with Cell-tracker Green/Red. 32G needles were used in (**a,b**), and 30G (inner diameter 0.15 mM) needles were used in (**c,d**). Scale bar 200 μm.

To demonstrate the further potential to be used in tissue engineering, fibers were assembled into different three dimensional structures. ESCs were stained with CellTracker Green or Red before encapsulated into hydrogel fibers. Tubular structures (Fig. 4a,b) made of helical coil or parallel coils were assembled by simply reeling the DNH fibers. Fibers were verified to be stiff enough to endure the reeling procedure manually without crack or degradation. Tightly firmed knots (Fig. 4c,d) were knitted with tweezers similarly. The tubular unit is similar to the structure of smooth muscle cells surrounding endothelial tubes^30^, suggesting that the orientation of cells within a 3D tubular structure could be controlled after the reeling process. Onoe *et al.* co-cultured HepG2 and NIH/3T3 cells within similar helical coil tube and found that intercellular communication between the different cell types can be regulated in the assembled three dimensional structures^1^. Knitted fabrics have also been used as skeleton to provide structural support for the other matrix with proper biological environment for tissue growth^31^. Ouyang *et al.* knitted hESC-encapsulated fibers to a sponge scaffold and used for tendon and ligament regeneration^32^.

In addition, combined with the weaving technology, these fiber-based spatially constructs would be able to mimic the intrinsic fabric-like network tissues such as neuronal pathways *in vivo*^33^. Thus we believe the proposed technology will be an attractive method for tissue engineering and regenerative medicine.

### Viability and Expression of Pluripotency Markers of the Encapsulated Embryonic Stem Cells

**Figure 5:**
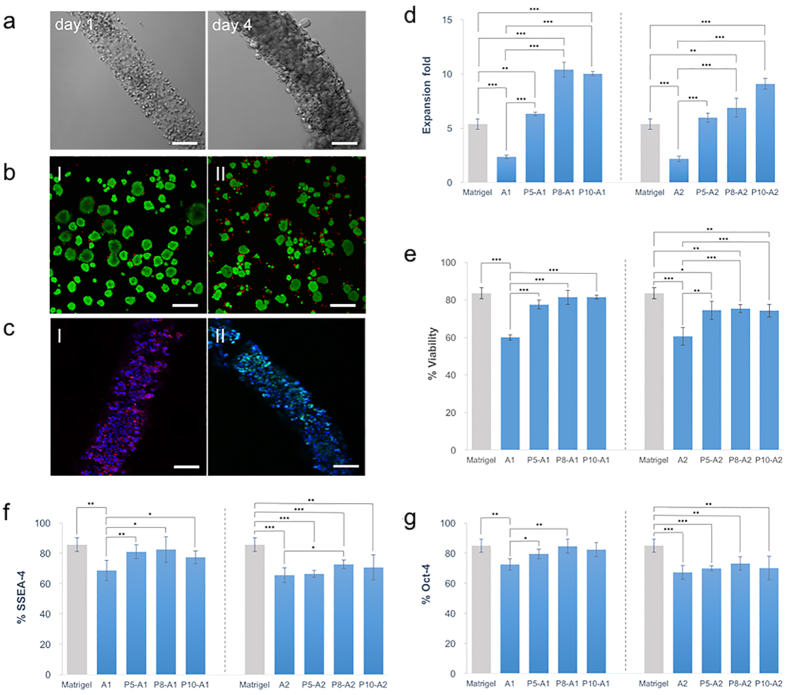
Cellular proliferation, viability and expression of pluripotency markers encapsulated in DNH. (**a**) ESCs proliferated in DNH during 4-day culture. Scale bar 100 μm. (**b**) Live/dead staining images of ESCs encapsulated in (I) P8-A1 and (II) A-1. Scale bar 200 μm. (**c**) Immunostaining images of (I) SSEA-4 and (II) Oct-4. Scale bar 200 μm. (**d**) Expansion folds comparison (**e**) Viability comparison (**f**) Expression level of SSEA-4 (**g**) Expression level of Oct-4 of ESCs cultured in different hydrogel after 4-day culture. The results are illustrated as the mean ± s.d. of five independent samples. *Indicates p < 0.05, **Indicates p < 0.005, ***Indicates p < 0.001.

To evaluate whether cells encapsulated in DNH were provided with suitable living condition, proliferation, viability and pluripotency were measured. ESCs cultured on Matrigel coated surface were tested as an additional control. The statistic results showed that by combining PNIPAAm-PEG, the proliferation rate of stem cells increased significantly in the DNH matrix (Fig. 5a,e). The expansion fold of cells encapsulated in P8-A1/P10-A1 reached up to 10.39/10.02 after 4-day culture while only 5.38 folds on Matrigel coated surface and 2.36 folds in 1% alginate. Considering the match of mechanical strength, we took a closer look at the expansion rate of cells in A1 and P8-A2. ESCs proliferated to 6.89 folds in P8-A2, almost tripled the data of A1 (merely 2.36 folds). We also accessed the viability of ESCs encapsulated in different matrix. Live/dead staining revealed that there was no significant difference between cells within P5-A1/P8-A1/P10-A1 and on Matrigel coated surface while there was a decline within A1 (Fig. 5b,f). Viability of cells decreased with higher concentration of alginate as a whole. Although combing PNIPAAm-PEG may decrease the mechanical strength (unfavorable to cell handling), it can be remedied by use a higher concentration of alginate with PNIPAAm-PEG added, which giving a much better extracellular matrix for ESCs to grow.

Furthermore, we investigated whether the pluripotency of stem cells was effected by the DNH system. The expression of stage-specific embryonic antigen-4 (SSEA-4) and octamer-binding transcription factor 4 (Oct-4) were tested. SSEA-4 is a glycolipid carbohydrate epitope that is expressed upon the surface of ESCs and Oct-4 is a protein that in humans is encoded by the POU5F1 gene. SSEA-4 and Oct-4 are critically markers involved in the self-renewal of undifferentiated ESCs. They have been widely used to represent pluripotency as expression of these markers reduced sharply in differentiated cells. Immunostaining showed there was no significant difference between the expression of SSEA-4 and Oct-4 on Matrigel coated surface and in P5-A1/P8-A1/P10-A1 (Fig. 5f,g). The uniform expression of both pluripotency marker Oct-4 and SSEA-4 remained high. Up to 82.46% expressed SSEA-4 and 84.66% of cells expressed Oct-4 in P8-A1while only 43.68% expressed SSEA-4 and 68.73% expressed Oct-4 in A1. Consistent with the results of proliferation and cell viability, higher concentration of alginate (2%) is not conductive to keep pluripotency of cells. The ability to maintain the pluripotency of embryonic stem cells is crucial otherwise it couldn’t differentiate into specialized lineages in tissue engineering or regenerative medicine^34^.

An additional 12 days culture (three passages) was conducted to investigate the influence of the processing conditions on stem cells. Cells were released, passaged and re-encapsulated into the DNH every 4 days. Live/dead staining revealed high viability (81.13%) after three passages. Immunostaining showed 85.27% expressed SSEA-4 and 81.96% cells expressed Oct-4. There was no significant difference between one passage and three passages. After going through these processing conditions, cells still maintain relative high pluripotency. It’s reasonable to confer that gel matrix provides more appreciate extracellular environment after combing PNIPAAm-PEG, which could guarantee high expansion rate, cell viability and pluripotency. Since stem cell is an extremely intricate system, physical, chemical and mechanical effectors in ECM are too great to define^35^. Influences on stem cells could be attributed to variables like topography^36^, mechanical strength^37^ and chemical compositions^38^. From the perspective of chemical composition, we hypothesized that the highly lipophilic environment at gel state of PNIPAAm-PEG and porous morphology could provide efficient niches comparing with pure alginate bulk which lack of proper binding sites.

### Degradation of DNH Fibers

Before using to subsequent pathological researches and drugs screening, cells may need to be released from matrix. In this proposed system, cells encapsulated in DNH fibers can be released out through different ways. Either component in DNH reaches a relative high level, the gel matrix could be destroyed by selective degrading this part and cells can be released.

**Figure 6:**
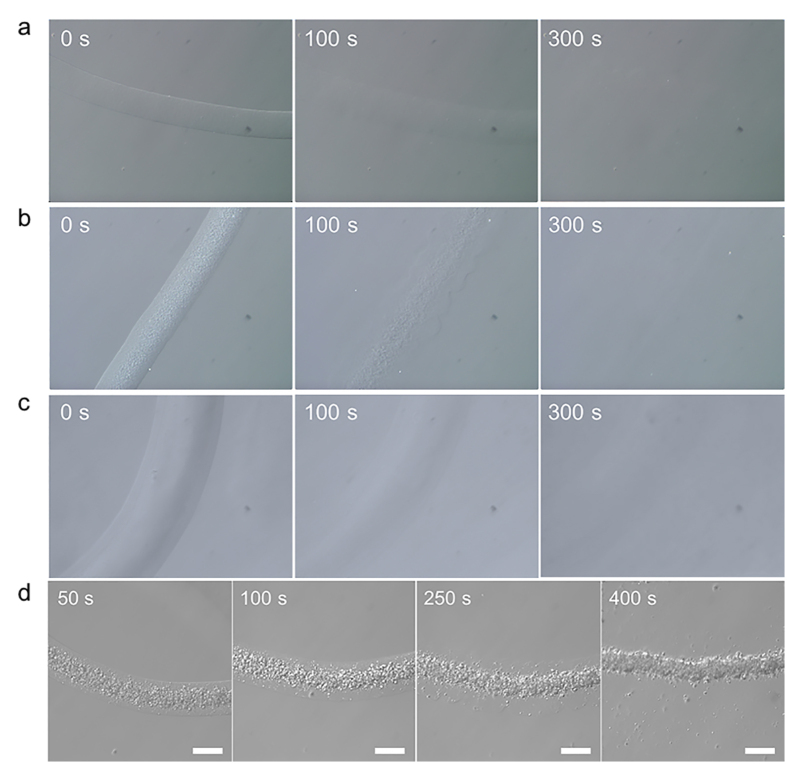
Different degradation methods of the DNH fibers. The DNH possessed specific quick responses to either (**a**) alginate lyase (0.4 mg mL^**−1**^), (**b**) EDTA (20 mM) or (**c**) low temperature (0–4 °C). (**d**) Cells can be released from the hydrogel fibers in about 5 min. Scale bar 200 μm.

In some cases, matrix with relative high mechanical strength is preferred such as bone tissue engineering^39^. In order to provide higher mechanical strength, the concentration of alginate should be increased. Therefore, the framework will be destroyed by degrading alginate in the DNH fibers. After culturing for 4 days in P5-A1, the alginate template used to support the fibers can be selectively dissolved by adding EDTA or alginate lyase. Figure 6a,b and Supplementary Movies S1 and S2 showed that the DNH possessed specific quick responses (less than 5 minutes) to both EDTA (20 mM) and alginate lyase (0.4 mg mL^−1^). However, like most of other materials, degradation of alginate needs to add exogenous reagents into the culture media, which may bring uncertain factors to some chemical sensitive system. As PNIPAAm-PEG is a thermo-responsive hydrogel, DNH fibers will melt in cold medium by putting into refrigerator under the circumstances that high proportion of NIPAAm-PEG is combined. Matrix with this degradation method suits for cells that prefer soft matrix to grow. As illustrated in Fig. 6c, P15-A1 fibers gradually melt into cold medium as expected. Through the above methods, matrix around the encapsulated cells can be removed. Microscope images shown in Fig. 6d demonstrated that the cells remained in the original position during the whole degradation process and didn’t float away with alginate. After the alginate temple was removed, the gel framework was destroyed, leaving the fibers containing only cells. It is a valuable improvement as it avoids inference caused by extracellular matrix.

## Discussion

Artificial reconstruction of functional three dimensional micro-tissues is an important technology which could contribute to the development regenerative medicine^40^. Approaches to reconstruct spatially organized and heterogeneous micro-tissues *in vitro* is a critical issue for the realization of actual applications^41^. In the mean time, development of new extracellular matrix for stem cell culture is expected to solve various challenges such as limited cell sources and immunologic rejection of allogeneic cells^42^.

Among different tissue building blocks, fibers have attracted lots of interest owing to their unique merits. Fibers could be used as basal building blocks for mimicking hierarchical fabric structures, such as vascular grafts^43^, nerve networks^44^, muscle fibers^45^ and tendons^32^. Besides, fibers are usually easy to fabricate, handle and assemble from the aspect of bioengineering. In the past decades, various methods have been developed to fabricate fibers. Electrospinning, wetspinning and microfluidic spinning are three of the most widely used methods. In this work, we proposed a simple method using microfluidic pneumatic dispensing system to prepare fibers with tunable and controlled diameter (Fig. 1). Pre-gel with cells was extruded into warm cross-linker solution. In the mean time, cells were fixed during this process. The gel matrix protected cells from the damage of sheer strength to some extent. According to the linear equation between practical diameter and inner diameter of corresponding needle, fibers with diameters ranging from 40–2000 μm were prepared by simply altering the specification of blunt needles. Moreover, fibers were verified strong enough to be assembled into different fabric structures like helical coil and knots using reeling or knitting methods (Fig. 4). Although electronicspinning is able to prepare fiber-based materials at nanoscale, serious damages are inevitable when the cell suspension is directly electrospun to substrates^10^. One the other hand, microfluidic spinning offers milder conditions and more opportunities in construction design^11^. Nonetheless, sophisticated equipment used in the process of lithography, especially overlay, well-trained specialists are indispensable. Combined with weaving technology, these fiber-based spatially constructs may have extensive use in tissue engineering and regerative medicine mimicking the inherent structures *in vitro*.

During the last decades, materials used in fiber-based technology have been extensively developed. Generally, materials for fiber formation need to have good biocompatibility, enough mechanical strength and able to be continuously manufactured^46^. One of the most widely used material is alginate, a natural-derived hydrogel with good cell compatibility usually. Nonetheless, performance is not satisfied when it comes to stem cells^27^. In this work, we combined a synthetic thermosenstive hydrogel PNIPAAm-PEG into alginate, forming a double-network hydrogel with tunable mechanical strength and attractive biocompatibility. As shown in Fig. 3, stiffness can be tuned by altering the amount of each component in the DNH. While alginate benefits the mechanical strength, PNIPAAm-PEG do favor to the flexibility. Combination of these two components overcame both the brittleness of alginate and low mechanical strength of PNIPAAm-PEG. We noticed that different from most double-network systems, the resulting matrix didn’t exhibit higher mechanical properties than alginate. The relative amount of Ca in pure bulk and DNH were tested to estimate the crosslinking degree of alginate. Results illustrated that incorporating PNIPAAm-PEG interfered with the crosslinking process of alginate. We hypothesize that the increasing viscosity and decreasing pore sizes (Fig. 2) simultaneously hindered the diffusion efficiency of Ca^2+^, reducing the actual amount of cross-linked alginate in DNH. In addition, biocompatibility of different hydrogels was evaluated. Among the six different formulation, P8-A1 is considered as the optimal matrix. Results showed that the cells encapsulated into P8-A1 exhibited significant higher expansion fold comparing to cells in alginate and on Matrigel coated surface (Fig. 5). The capacity of scalable expansion is critical to solve the urgent shortage of stem cell resources. Besides, live/dead staining and immunostaining were also carried out to evaluate whether DNH matrix and processing procedures interfered with the viability and pluripotency of stem cells or not. Results revealed that there was no significant difference between cells in P8-A1 and on Matrigel coated surface. By contrast, cells in alginate showed an obvious decrease. As stem cell is a complex system and various factors may influence on the stem cell fate, it is hard to clarify the specific reason for the biocompatibility increase. From the perspective of chemical composition and morphology, we assume that the highly lipophilic environment at gel state of PNIPAAm-PEG and highly porous morphology offered cells with more sites to attach and particularly benefited the exchange of both nutrients and metabolic wastes.

Additionally, both of the crosslinking and degradation conditions are mild in the proposed system^27^,^47^. The system allowed cell release by thermal degradation and enzymatic or calcium chelation. Different from many synthetic matrix, cell-damages caused by UV light or residual initiator are avoided^26^. An additional 12 days culture (three passages) was implemented to support this claim. Results showed that no significant difference occurred between after three passages, proving that the processing conditions have no negative effects on stem cells.

Despite the advantages mentioned above, there are still some further work need to be studied. Efforts should be paid to increase the spatial resolution under precise control. Different type of cells involved in relative disease models should be tested. As one practical application, HUVECs were cultured in the DNH fibers. Immunostaining showed extensive expression of VE-Cadherin (Fig. S3 and Supplementary Method 2), a class of indispensable protein for proper vascular development through controlling of cohesion and organization of the intercellular junctions^48^. This result preliminary confirmed the application prospect of the proposed system. Differentiation of stems cells within the DNH into multiple lineages and performance of the system after transplantation *in vivo* also need to be evaluated in the future work.

## Conclusion

We proposed a double-network hydrogel composed of alginate and PNIPAAm-PEG with tunable mechanical performance and biocompatibility for the three dimensional assemble of stem cells-encapsulated fibers. The combination of these two material overcame both the brittleness of alginate and low mechanical strength of PNIPAAm-PEG. Fibers with diameter ranging from 40–2000 μm have been parepared simply by altering the specification of blunt needle. Besides, the double-network hydrogel fibers can be assembled into fabric constructs such as helical coils and knots using reeling and knitting technology. We further studied the cell behavior encapsulated within the double-network hydrogels. The optimal matrix P8-A1 exhibited significant higher expansion fold comparing to cells in alginate or on Matrigel coated surface. The capacity of scalable expansion is beneficial to relieve the urgent shortage of stem cell resources. Besides, live/dead staining and immunostaining showed there was no significant difference between cells in P8-A1 and on Matrigel coated surface. Moreover, the proposed system allowed cell release by thermal degradation and enzymatic or calcium chelation. A three passages culture were conducted to prove that the processing conditions having no significant effects on stem cells. To conclude, We have good reasons to believe that this versatile system may further contribute to tissue engineering and regenerative medicine.

## Methods

### Fabrication of DNH fibers

Pre-gel solution of alginate (average molecular weight 120,000–190,000 g mol^−1^, 4% wt/vol, dissolving in ddH2O) and PNIPAAm-PEG (Mebiol Gel, average molecular weights of is over 100,000, content of PEG is 38 wt/wt, 20% wt/vol, dissolving in E8 medium) were mixed at different ratio. All the solutions were filtered through filter membrane (0.22 μm) before use. Cells were encapsulated into the composite materials thoroughly on ice if needed. The mixture was kept on ice during the whole process to prevent gelation. Microfluidic pneumatic dispensing system was used to fabricate DNH fibers. Before loading cells into the microfluidic device, all the luer stubs, connectors and tubing were sterilized by autoclaving. After loading cells into the stub, the device was reset. Then air pump was turned on to extrude fibers. Once the pre-gel solution was extruded into warm crosslinking solution made of CaCl_2_ (100 mM) and sucrose (3% w/w), it went through a sol-gel transition immediately and cells were encapsulated into the fiber at the same time. The suspended solidified fibers were transferred into culture medium and put back into CO_2_ incubator.

### Degradation of DNH fibers

The DNH fibers can be gradated through three different pathways. 1) Alginate lyase (4 mg mL^−1^) in DPBS (−) was added at 1:10 (v/v) to the culture medium where fibers dispersing for 5 min at 37 °C. 2) Fibers were immersed into EDTA (20 mM) for 5 min at 37 °C. 3) Fibers that dispersing in culture medium was put into refrigerator for 5 min.

### Morphology

Morphology and microstructure of different hydrogel were examined using Scanning Electron Microscopy (Quanta 200, FEI). Before observation, the hydrogels were frozen in liquid nitrogen and freeze-dried (Christ) at −55 °C for 24 h.

#### Rheological characterization

Rheological experiments were carried out on an AR-G2 rheometer (TA Instruments) equipped with temperature controller. Tests were performed in 8 mm parallel-plate geometry with a gap size of 0.5 mm. In order to find the linear viscoelastic region, oscillatory strain sweep from 0.01–1000% was conducted at 25 °C with a fixed frequency of 1 Hz. Temperature-ramp tests were performed at a fixed frequency 1 Hz and strain 0.5%. Shear storage modulus (G’) and shear-loss modulus (G”) were measured from 4–40 °C at a rate of 4 °C min^−1^.

### Cell culture

Human embryonic stem cell lines H9 was purchased from CELLAPY. H9 were cultured on six-well plates coated with Matrigel in E8 medium. Cells were passaged every 4–5 days with dissociation agent EDTA (0.5 mM). In order to transfer from six-well plate to hydrogel, H9 clones were dissociated into single cells at 37 °C for about 5–7 min, and then encapsulated into DNH at desired density, culturing in E8 medium supplied with ROCK inhibitor (10 μM) for the first 24 h. In the 12 days culture, cells were released from the DNH using alginate lyase and lower temperature. After dissociation into single cells with EDTA, cells were counted and re-capsulated into DNH. Passage number of cells used in this paper were among 48–65.

### Staining and Imaging

To assess the proliferation of cells embedded in different hydrogel, cells were released and stained with Hoechst 33342 after 4-day culture with an initial density of 10^7^ mL^−1^. Cell suspension was added onto a hemocytometer and observed with Olympus IX71 microscopy. Software Image J. was used to count cells.

To evaluate cell viability, ESCs encapsulated in different DNH were cultured for 4 days, released and dissociated into single cells before stained with live/dead cell viability assay and imaged with confocal laser-scanning microscopy (Nikon, A1RSi). Software Image J. was used to count cells.

To assess the expression level of pluripotency marker Oct-4 and SSEA-4, ESCs encapsulated in different DNH were cultured for 4 days and released and dissociated into single cells before immunostaining. Cells were fixed with paraformaldehyde (PFA) (4% wt/vol) for 1 h, permeabilized with Triton-X (0.1%, vol/vol) for 10 min, and blocked with bovine serum albumin (BSA) (1%, wt/vol) for 90 min to eliminate nonspecific bindings. Then cells were incubated with primary antibodies Oct-4 (1:200)/SSEA-4 (1:100) both in BSA (1%, wt/vol) solution for 1 h at 37 °C in incubator. After extensive washing with DPBS, cells were incubated with secondary antibodies (1:200 and 1:500 respectively) in BSA (1%, wt/vol) solution for 30 min at 37 °C. Hoechst 33342 (1:1000) in DPBS was carried out for nucleus staining. Cells were washed with DPBS for three times before imaging with Olympus IX81 microscopy. Software Image J. was used to count cells.

For the fiber-based stainning, processes of cell release and dissociation are cancelled. Confocal laser-scanning microscopy (Nikon, A1RSi) with excitation wavelength of 488 nm or/and 561 nm was used for cell imaging.

### Waving and reeling of fibers

Before use, the glass tube was coated with low melting-point agarose (3%). Cells were stained with CellTracker Green or Red before encapsulated into hydrogel. Fibers extruded in E8 culture medium were picked up with the agarose-coated glass tube in the air, and then reeled up to make a helical structure. Then the helical structure was dipped into the low-melting agarose again to prevent the structure from collapsing. Finally, the glass tube was removed, the structure was released into culture medium and put back into incubator. To fabricate a knot structure, tweezers were the only things that needed. The constructs were observed with confocal laser-scanning microscopy (Nikon, A1RSi).

### Cell release

In order to remove the hydrogel in the fibers, alginate lyase (4 mg mL^−1^) in DPBS (−) was added at 1:10 ratio to the medium where fibers were dispersing. Then fibers were incubated for 5 min to enzymatically digest the Ca^2+^-alginate at 37 °C, followed by 15 min at 4 °C to melt the thermo-sensitive hydrogel thoroughly. Finally, fibers consisting of only cells were obtained.

## Additional Information

**How to cite this article**: Liang, Z. *et al.* Double-Network Hydrogel with Tunable Mechanical Performance and Biocompatibility for the Fabrication of Stem Cells-Encapsulated Fibers and 3D Assemble. *Sci. Rep.* **6**, 33462; doi: 10.1038/srep33462 (2016).

## Supplementary Material

Supplementary Information

Supplementary Movie S1

Supplementary Movie S2
