# Supplementary material for: Double-Network Hydrogel with Tunable Mechanical Performance and Biocompatibility for the Fabrication of Stem Cells-Encapsulated Fibers and 3D Assemble
Source: Sci Rep. 2016 Sep 15;6:33462. doi: 10.1038/srep33462 (PMC5024157; doi:10.1038/srep33462)
Supplement: Supplementary Information [file srep33462-s1.doc]

**Double-Network Hydrogel with Tunable Mechanical Performance and Biocompatibility for the Fabrication of Stem Cells-Encapsulated Fibers and 3D Assemble**

**Zhe Liang, Chenguang Liu, Lili Li, Peidi Xu, Guoan Luo, Mingyu Ding and Qionglin Liang***

Department of Chemistry, Tsinghua University, Beijing, 100084, China

* [liangql@tsinghua.edu.cn](mailto:liangql@tsinghua.edu.cn)

**Supplementary information**

**Figure S1. Fourier transform infrared (FTIR) spectroscopy** was used to confirm whether chemical interactions happened between the two polymers. FTIR spectra of the both DNH and two pure components are shown.


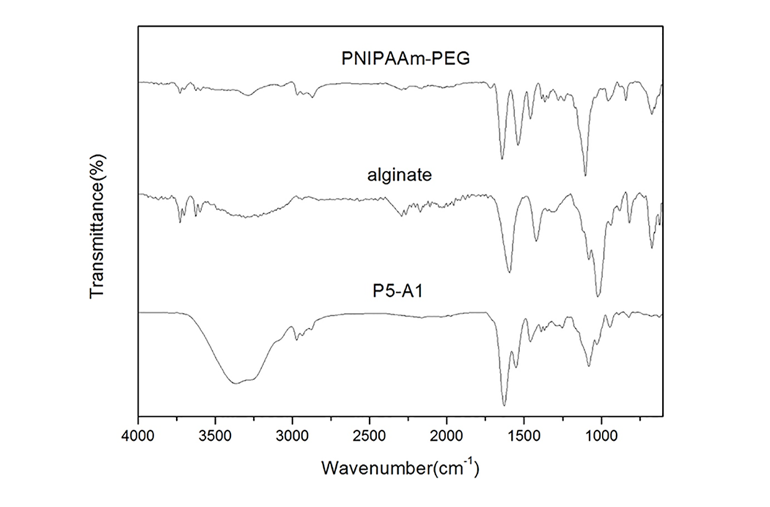


The peaks at 1594, 1420, 944 and 821 cm^-1^ in pure alginate FTIR spectra were associated to the asymmetric stretching and symmetric COO^-^ stretching vibration, the manuronic and gluronic acid functional groups, respectively. The peaks at 1643 cm^-1^ and 1539 cm^-1^ were the absorption of amide I and amide II in pure PNIPAAm-PEG FTIR spectra. All these characteristic peaks showed up in the spectra of DNH and no peaks suggesting chemical interactions were observed. The presence of the broad-band at 3200–3700 cm^–1^ in DNH can be explained from two aspects. Besides O-H and N-H contributed part of the peak, the changes in microstructure played an important role. The porous microstructure of the DNH leaded to better hygroscopicity comparing to the lamellar structure of the two pure components. However, it should be noted that although no covalent bonding was observed between alginate and PNIPAAm-PEG, possible non-covalent interactions might still had happened during the process.

**Figure S2. Temperature-ramp rheological analysis of alginate was tested from 4 to 40 °C.** Frequency was fixed at 1 Hz and strain was fixes at 1 %.


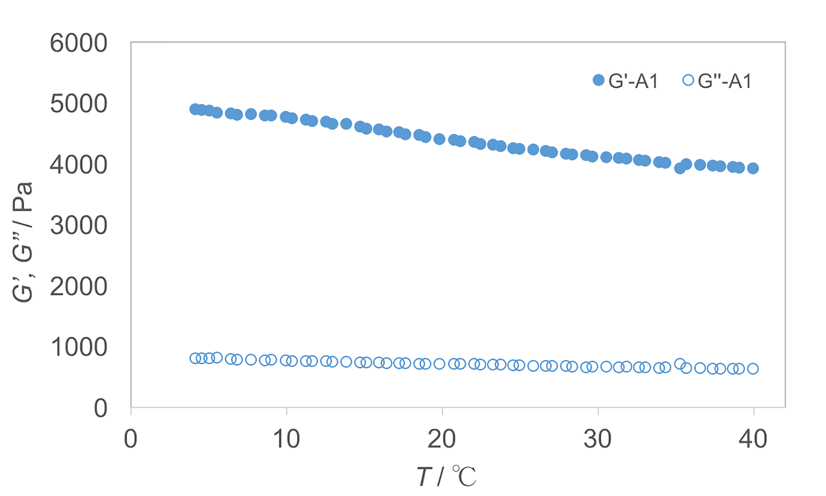


**Figure S3. The expression of VE-Cadherin in HUVECs fiber.** Scale bar 200 μm.


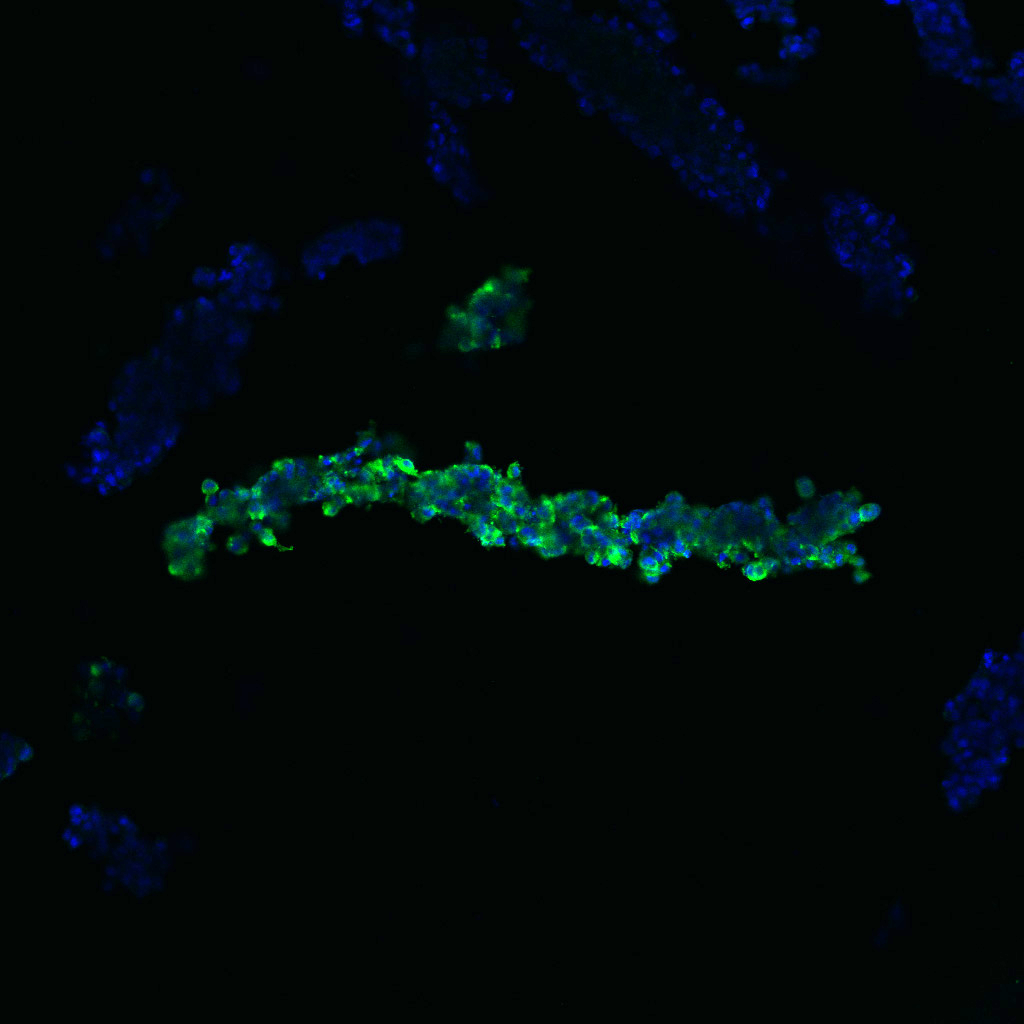


**Supplementary Method 1. Calculation of the crosslinking degree in different matrix.**

Equal volume of prepared A1 and P10-A1 hydrogel were loaded into dialysis bags with molecular weight cutoffs 14000 and cross-linked as mentioned in **Fabrication of DNH fibers** section. Then the hydrogels were washed with ddH_2_O three times and put into a lyophilizer to remove the containing water. Samples were weighted before loading into ICP-OES (Vista MPX, Varian) for Ca analysis. Test results showed that the relative amount of Ca in A1 was 7.89% (wt/wt) while 0.67% in P10-A1. The crosslinking degree was converted and calculated as follows.


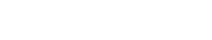


where
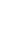
 is the actual mass of Ca which was cross-linked in the matrix,
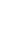
 is the theoretical mass of Ca in fully cross-linked condition.

In matrix A1,
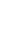
 and
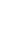
 is calculated as follows.


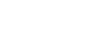


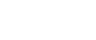


where
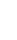
 is the relative amount of Ca read out from ICP-OES directly,
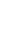
 is the theoretical amount of Ca in fully cross-linked condition. W is the mass of Ca-alginate in the sample. In systems without PNIPAAm-PEG, it is equal to the sample weight.
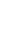
 is calculated to be 10.26% assuming that the complex ratio between alginate and Ca^+2^ is 2:1. Based on the equations above, the crosslinking degree of A1 is calculated to be 78.9%.

As to P10-A1, algorithm of
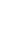
 is same with that of A1 while
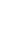
 is calculated as follows.


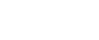


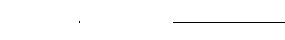


where W’ is the mass of Ca-alginate in P10-A1. V is the volume of P10-A1 solution.
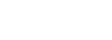
 is molecular weight of the structural unit of Ca-alginate based on the complex ratio 2:1.
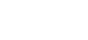
 is molecular weight of the structural unit of alginate.
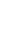
 is 10.26% as mentioned before. Based on the equations above, the crosslinking degree of P10-A1 is calculated to be 49.9%.

**Supplementary Method 2**

Human umbilical vein endothelial cells (HUVECs) were were maintained in culture flasks in DMEM culture medium supplied with 10% fetal bovine serum in an incubator with 5% CO_2_ at 37°C. Cells were trypsinized from the culture flask before re-suspended into P8-A1 at density of 10^7^ mL^-1^. Cell fibers were prepared as mentioned in before. Then the HUVEC fibers were fixed with paraformaldehyde (PFA) (4 %, wt/vol) for 30 min, permeabilized with Triton-X (0.1 %) in DPBS for 10 min and blocked with bovine serum albumin (BSA) (1 %, wt/vol) in DPBS for 60 min to eliminate nonspecific bindings. And then the fibers were incubated with primary antibodies VE-Cadherin (1:200) in 1% BSA for 1h at 37 °C in incubator. After extensive washing with DPBS, the fibers were incubated with secondary antibodies (1:200) in 1% BSA for 30 min at 37 °C. Hoechst 33342 (1:1000) in DPBS was carried out for nucleus staining. The fibers were washed with DPBS before imaging with confocal laser-scanning microscopy (Nikon, A1RSi) with excitation wavelength of 488 nm.

**Movie S1. Degradation procedure through 0.4 mg mL^-1^ alginate lyase. Recorded at 100X speed.**

**Movie S2. Degradation procedure through 20 mM EDTA. Recorded at 75X speed.**
